# Retroperitoneal urothelial carcinoma arising after bladder diverticulectomy: a case report

**DOI:** 10.1186/s12894-023-01266-x

**Published:** 2023-05-10

**Authors:** Akira Ohtsu, Seiji Arai, Yuji Fujizuka, Reon Fukuda, Keisuke Hori, Yuki Morimura, Rintaro Kawahara, Takuya Shiraishi, Hiroomi Ogawa, Yoshiyuki Miyazawa, Masashi Nomura, Yoshitaka Sekine, Hidekazu Koike, Hiroshi Matsui, Kazuhiro Suzuki

**Affiliations:** 1 Department of Urology, Gunma University Hospital, 3-39-15, Showa-Machi, Maebashi, Gunma Japan; 2 Department of General Surgical Science, Graduate School of Medicine, Gunma University, 3-39-15, Showa-Machi, Maebashi, Gunma Japan

**Keywords:** Urothelial carcinoma, Diverticulectomy, Multiparametric MRI

## Abstract

**Background:**

Urothelial carcinoma arises from the inner urothelial membrane of the renal pelvis, ureter, and bladder and often causes macrohematuria. Here, we report a rare case in which the patient developed non-symptomatic urothelial carcinoma anatomically outside the bladder wall 17 years after bladder diverticulectomy.

**Case presentation:**

An 82-year-old male patient previously underwent gastrectomy for stomach cancer and partial hepatectomy for intrahepatic cholangiocarcinoma. Follow-up computed tomography revealed a tumor in the retroperitoneal space, where a bladder diverticulum was removed 17 years earlier. Multiparametric magnetic resonance imaging suggested that the tumor was malignant with rectal invasion. Subsequent computed tomography-guided percutaneous biopsy revealed that the tumor was urothelial carcinoma. The patient underwent two courses of neoadjuvant chemotherapy followed by pelvic exenteration with pelvic lymph node dissection. He is currently receiving adjuvant therapy with an immune checkpoint inhibitor and has had no recurrence for 3 months.

**Conclusions:**

Multiparametric magnetic resonance imaging is a helpful tool for predicting both tumor malignancy and invasion before a pathologically confirmed diagnosis. Although this case is rare, urologists should be aware of the occurrence of urothelial carcinoma after bladder diverticulectomy in cases of incomplete resection of the diverticulum.

**Supplementary Information:**

The online version contains supplementary material available at 10.1186/s12894-023-01266-x.

## Background

A urinary bladder diverticulum is an out-pouching of the bladder mucosa through a weak bladder muscle, either congenital or acquired, which may be complicated by inflammation, calculus, infection, and malignancy [1]. If malignancy or inflammation recurs, diverticulectomy could become an option. Here, we report an exceptional case of a patient who developed urothelial carcinoma anatomically outside the bladder wall 17 years after a bladder diverticulectomy.

## Case presentation

**Fig. 1:**
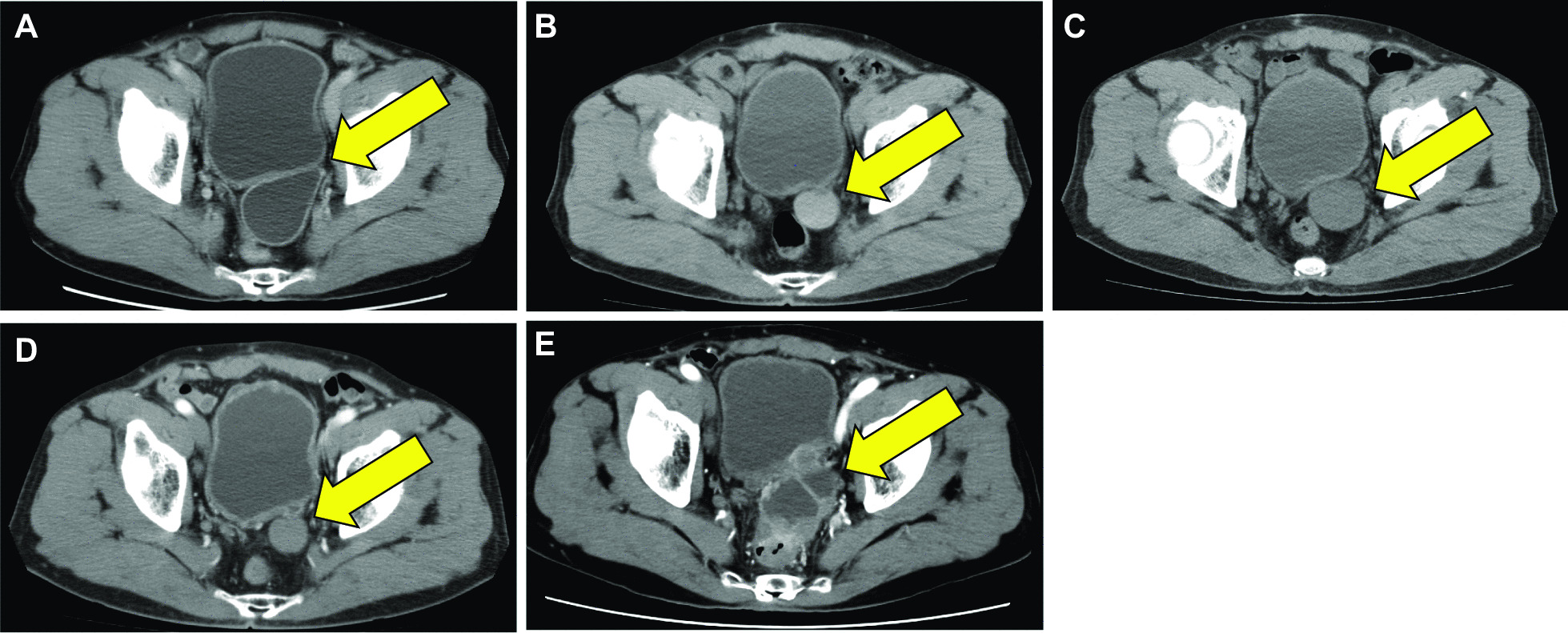
CT images before (**A**), 8 months (**B**), 3 years (**C**), 8 years (**D**), and 17 years after (**E**) diverticulectomy. The yellow arrows indicate the diverticular area. CT, computed tomography

An 82-year-old male patient was admitted to our urology department with a retroperitoneal tumor. He received a bladder diverticulectomy at our urology department about 17 years ago (Fig. 1A). The previous medical record also revealed that he underwent gastrectomy for gastric cancer at the gastrointestinal surgery department 22 years ago and partial hepatectomy for intrahepatic cholangiocarcinoma at the hepato-biliary-pancreatic surgery department 2 years ago (Additional file 1: Fig. S1). He received all surgeries at Gunma University Hospital. After the diverticulectomy, he has undergone clean intermittent catheterization for urinary retention. Eight months after the surgery, computed tomography (CT) revealed a high-density, unenhanced retroperitoneal mass with a smooth margin near the previous diverticulectomy site (Fig. 1B). At that time, the mass was diagnosed as a non-malignant tumor (e.g., hematoma). Follow-up CT series showed that the tumor density became low, and the size occasionally grew and shrunk (Fig. 1C, D).

**Fig. 2:**
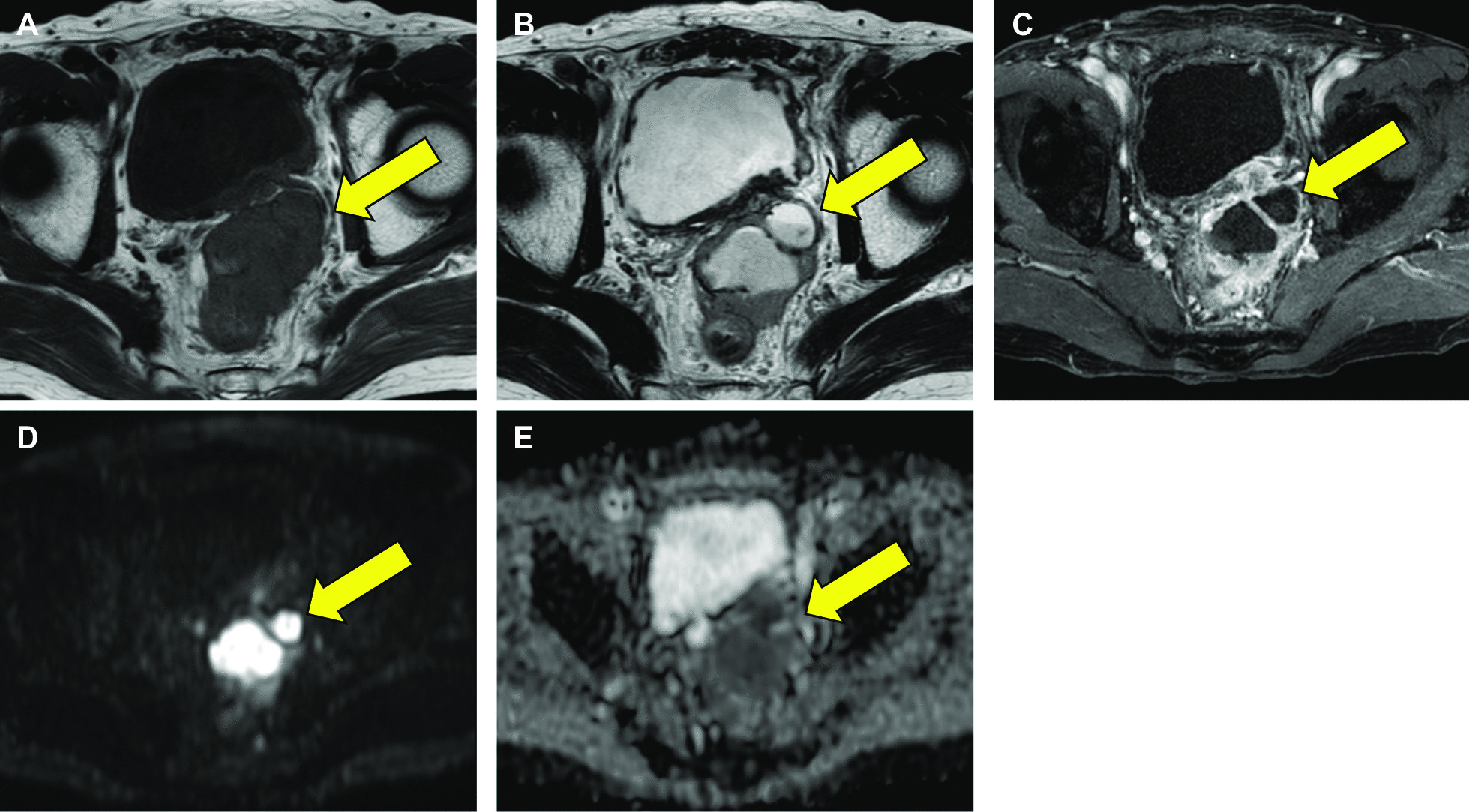
Contrast-enhanced MRI images: T1W (**A**), T2W (**B**), DCE (**C**), DWI (**D**), and apparent diffusion coefficient (ADC) map (**E**). The yellow arrow indicates the diverticular area. T1W, T1-weighted; T2W, T2-weighted; DCE, dynamic contrast-enhanced; DWI, diffusion-weighted image

**Fig. 3:**
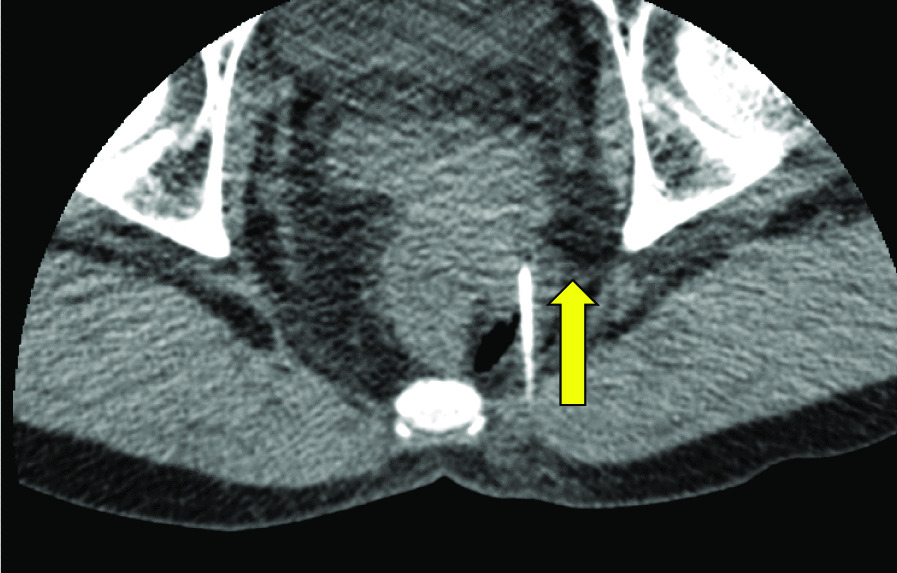
CT-guided percutaneous needle biopsy. The yellow arrow indicates the direction of the biopsy. CT, computed tomography

**Fig. 4:**
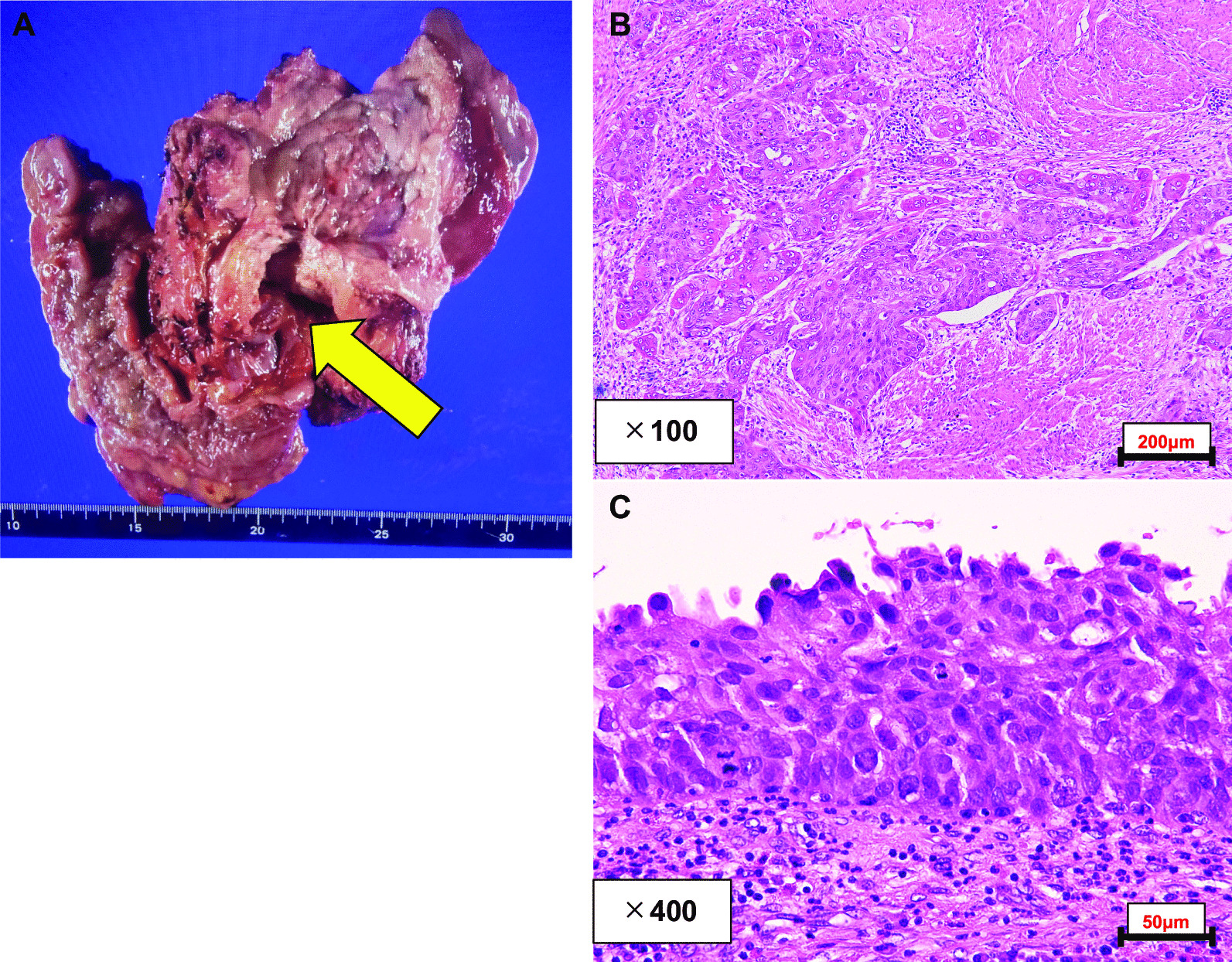
Surgically removed specimens (**A**). The diagnosis was urothelial carcinoma with squamous differentiation (**B**, **C**). The yellow arrow indicates carcinoma

Seventeen years after the diverticulectomy, CT revealed the enhanced tumor with an irregular margin in the same location, and the size of the tumor increased gradually over time (Fig. 1E). Tumor markers were within their respective standard ranges (prostate-specific antigen: 0.58 ng/ml, carcinoembryonic antigen: 1.9 ng/ml, carbohydrate antigen 19–9: 13 U/ml, and interleukin-2R: 532 U/ml). Cystoscopy did not show any tumor on the surface of the bladder membrane, and urine cytology did not reveal neoplastic findings. However, multiparametric magnetic resonance imaging (MRI) indicated that the tumor was malignant and had invaded the seminal vesicles and rectum (Fig. 2). Accordingly, CT-guided percutaneous biopsy was performed and revealed that the tumor was pathologically urothelial carcinoma (Fig. 3). Significantly, the previous surgical record revealed that part of the diverticulum with urothelial membrane had remained in the body postoperatively owing to the anatomical difficulty of complete resection, suggesting that the urothelial membrane remnant in the diverticulum developed urothelial cancer. The patient underwent two courses of neoadjuvant chemotherapy (gemcitabine and carboplatin) and subsequent pelvic exenteration with pelvic lymph node dissection. We performed combined laparoscopic transanal total mesorectal excision and open transabdominal surgery for tumor resection. The pathological diagnosis was invasive urothelial carcinoma with squamous differentiation and invasion of the seminal vesicles and rectum, pT4aN0M0, Stage IIIa (Fig. 4). The patient is currently receiving adjuvant therapy with an immune checkpoint inhibitor (nivolumab) and has had no recurrence for 3 months.

## Discussion and conclusions

Bladder diverticulum is managed conservatively for the patients without any symptoms or complications. However, surgical management can become the treatment option once they develop repeat urinary infections, bladder stones, or malignant tumors [1]. Among them, bladder cancer is of particular concern since the bladder diverticulum lacks the muscle wall beyond the mucosal layer, resulting in an increased risk for the extension of cancer cells outside the bladder.

This patient underwent bladder diverticulectomy to address repeat urinary tract infections. At the surgery, we initially attempted laparoscopic resection, but the extravesical approach to the diverticulum was anatomically difficult owing to the lateral vascular pedicle of the bladder. Therefore, we changed the approach and selected open surgery. We were able to remove the diverticulum with the intravesical approach; however, complete resection was difficult owing to the anatomical difficulty of approaching the bottom of the diverticulum. As a result, part of the diverticulum and the urothelial membrane remained in the body postoperatively. Because the cancer source appeared to be the urothelium, we assumed that the remaining urothelium in the body became malignant over time. Therefore, urologists should be aware of the occurrence of urothelial carcinoma after bladder diverticulectomy in cases of incomplete resection of a diverticulum.

Another possible surgical management for the bladder diverticulum could be transurethral endoscopic surgery, especially for those who previously received abdominal surgery or radiation. Since pre-surgical or radiation-induced adhesion makes it challenging to approach the bladder diverticulum in open or laparoscopic surgery, transurethral approach could be a better option. Several reports showed that transurethral fulguration of the urothelial membrane inside the diverticulum could shrink the bladder diverticulum over time [2, 3]. If this patient was treated with transurethral fulguration instead of open surgery, his bladder cancer could be found earlier or might not be developed later.

Retroperitoneal masses not arising from major solid organs are uncommon. Although there is no simple method of classifying retroperitoneal masses, a reasonable approach is to consider the masses as predominantly solid or cystic and to subdivide these into neoplastic and nonneoplastic masses. Cross-sectional imaging with CT or MRI is the backbone of the non-invasive characterization of retroperitoneal masses. CT provides better spatial resolution and is the best modality to detect calcifications. In contrast, MRI provides better soft tissue contrast and, importantly, allows more accurate local bladder cancer staging compared with CT [4]. Multiparametric MRI studies consist of anatomical T2-weighted images, functional diffusion-weighted images, and dynamic contrast-enhanced images. Imaging with multiparametric MRI has improved cancer diagnosis for bladder cancer [5]. In the present case, both cystoscopy and urine cytology were not helpful for the diagnosis because the tumor lacked continuity with the bladder. However, periodic imaging studies with CT showed a trend toward enlargement. Furthermore, MRI predicted tumor malignancy by decreased diffusion capacity and showed tumor invasion into the seminal vesicles and rectum. This case suggests that multiparametric MRI is more helpful than CT for distinguishing retroperitoneal masses between neoplasms and non-neoplasms. However, the use of MRI may be limited in some patients owing to safety concerns when metallic implants are present. Therefore, it is essential to identify potential safety hazards before performing MRI for retroperitoneal masses.

Imaging is not always sufficient to confirm the clinical diagnosis of a tumor, and histopathological assessment is indispensable, primarily when imaging cannot determine the malignancy of the tumor. Regarding tumor biopsy, ultrasound- or CT-guidance enables the safe procurement of samples for histological analysis [6, 7]. Notably, percutaneous biopsy of urothelial carcinoma might increase the risk of tumor tract seeding [8, 9]; tract recurrences often occur within 15 months. In contrast, in a study of 24 upper urothelial carcinomas with percutaneous biopsy, no tract seeding occurred in any of the cases [10]. In the present case, we performed a CT-guided percutaneous biopsy to reach a pathological diagnosis. Therefore, we will monitor the patient closely for tract seeding for at least 15 months. Given the tumor's location, a transrectal approach would have been a possible option for tumor biopsy [11], and considering that we performed subsequent pelvic exenteration, the effects of seeding might not have been a concern if we chose transrectal biopsy.

To the best of our knowledge, this is the first report on treating retroperitoneal urothelial carcinoma arising after diverticulectomy. If we had known about cancer earlier, surgical treatment with bladder and rectal preservation might have been possible. Even though the case is rare, urologists should be aware and consider the development of urothelial carcinoma after diverticulectomy.

## Supplementary Information

**Additional file 1. Supplemental Figure 1.** Resection specimen for partial hepatectomy.

## Data Availability

Not applicable.

## Abbreviations

CT: Computed tomography
MRI: Magnetic resonance imaging
