# Supplementary material for: Retroperitoneal urothelial carcinoma arising after bladder diverticulectomy: a case report
Source: BMC Urol. 2023 May 10;23:88. doi: 10.1186/s12894-023-01266-x (PMC10173469; doi:10.1186/s12894-023-01266-x)
Supplement: Supplementary file 1 — Additional file 1. Supplemental Figure 1. Resection specimen for partial hepatectomy. [file 12894_2023_1266_MOESM1_ESM.pptx]

## Slide 1
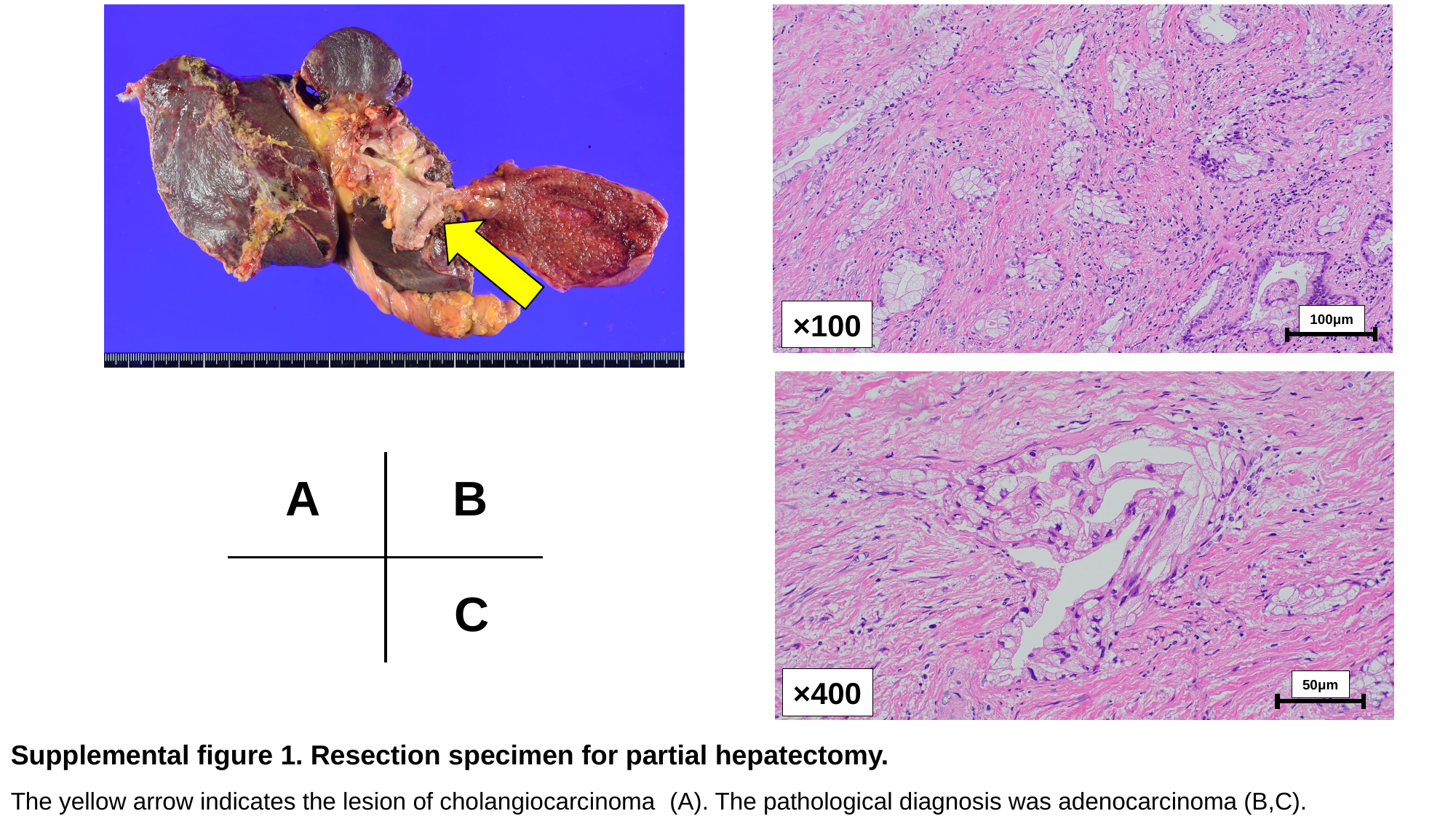

×100
100μm
B
A
C
×400
50μm
Supplemental figure 1. Resection specimen for partial hepatectomy.
The yellow arrow indicates the lesion of cholangiocarcinoma (A). The pathological diagnosis was adenocarcinoma (B,C).
